# Gapmer Antisense Oligonucleotides Suppress the Mutant Allele of *COL6A3* and Restore Functional Protein in Ullrich Muscular Dystrophy

**DOI:** 10.1016/j.omtn.2017.07.006

**Published:** 2017-07-08

**Authors:** Elena Marrosu, Pierpaolo Ala, Francesco Muntoni, Haiyan Zhou

**Affiliations:** 1 Dubowitz Neuromuscular Centre, Molecular Neurosciences Section, Developmental Neurosciences Programme, University College London, Great Ormond Street Institute of Child Health, 30 Guilford Street, London WC1N 1EH, UK

**Keywords:** collagen VI, congenital muscular dystrophy, dominant mutations, antisense oligonucleotide therapy, gapmer, allele-specific silencing

## Abstract

Dominant-negative mutations in the genes that encode the three major α chains of collagen type VI, *COL6A1*, *COL6A2*, and *COL6A3*, account for more than 50% of Ullrich congenital muscular dystrophy patients and nearly all Bethlem myopathy patients. Gapmer antisense oligonucleotides (AONs) are usually used for gene silencing by stimulating RNA cleavage through the recruitment of an endogenous endonuclease known as RNase H to cleave the RNA strand of a DNA-RNA duplex. In this study, we exploited the application of the allele-specific silencing approach by gapmer AON as a potential therapy for Collagen-VI-related congenital muscular dystrophy (COL6-CMD). A series of AONs were designed to selectively target an 18-nt heterozygous genomic deletion in exon 15 of *COL6A3* at the mRNA and pre-mRNA level. We showed that gapmer AONs can selectively suppress the expression of mutant transcripts at both pre-mRNA and mRNA levels, and that the latter strategy had a far stronger efficiency than the former. More importantly, we found that silencing of the mutant transcripts by gapmer AONs increased the deposition of collagen VI protein into the extracellular matrix, thus restoring functional protein production. Our findings provide a clear proof of concept for AON allele-specific silencing as a therapeutic approach for COL6-CMD.

## Introduction

Collagen-VI-related congenital muscular dystrophies (COL6-CMD) are the second most common diagnosis in congenital muscular dystrophies according to a recent retrospective review performed by our center on genetic studies in childhood neuromuscular diseases in the UK population.^1^ The collagen-VI-related myopathies, ranging from severe Ullrich congenital muscular dystrophy (UCMD) to mild Bethlem myopathy (BM) and intermediate clinical phenotypes, are caused by mutations in one of the genes that encode the three major α chains of collagen type VI: *COL6A1* (MIM*120220), *COL6A2* (MIM*120240), and *COL6A3* (MIM*120250).^2^, ^3^ The severe UCMD is an early-onset CMD variant that presents with slowly progressive muscle weakness, progressive joint contractures, and respiratory failure and the frequent development of severe scoliosis.^4^ Currently, there is no cure for COL6-CMD.

**Figure 1:**
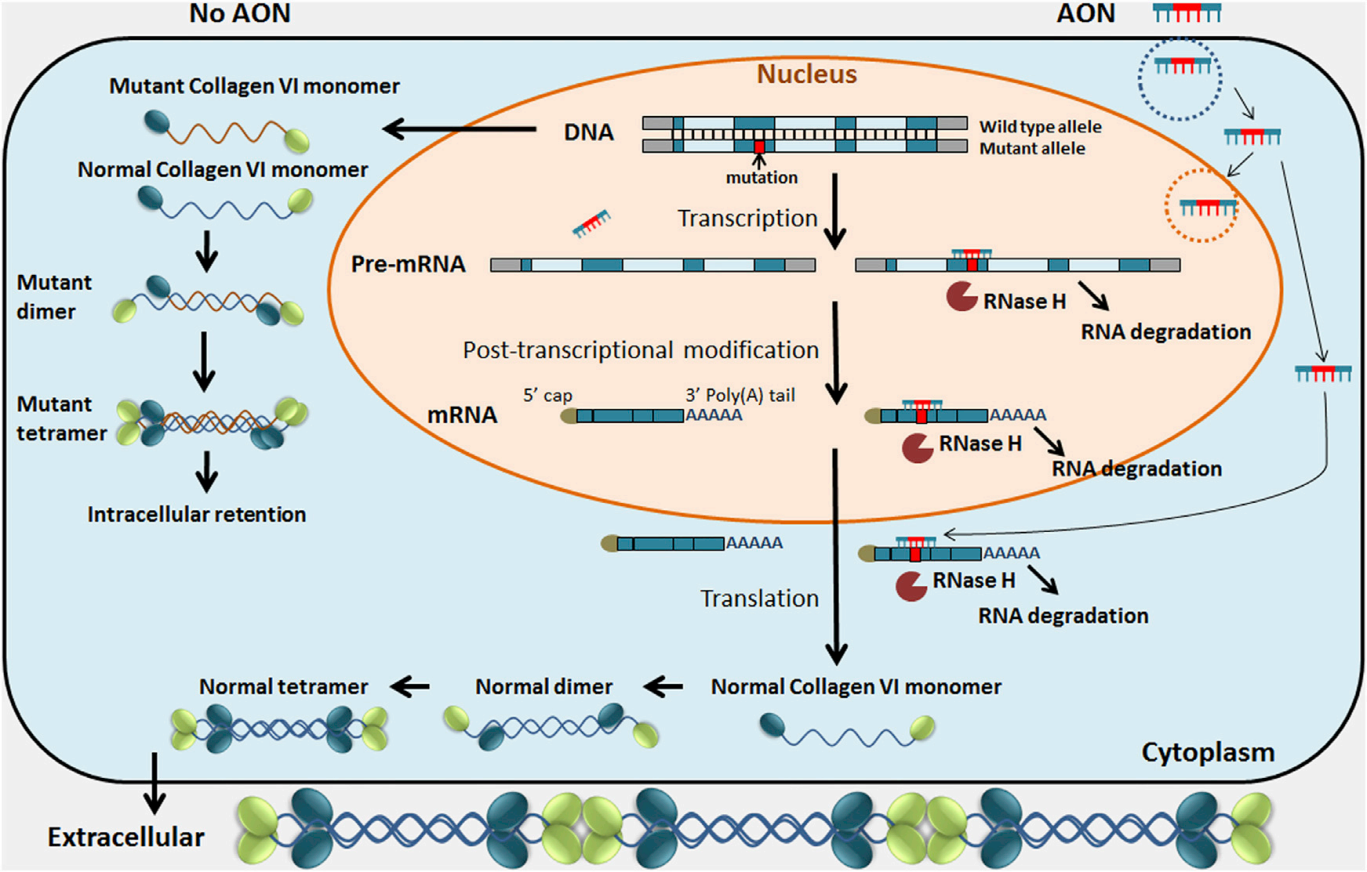
Schematic Diagram of Allele-Specific Silencing by Gapmer AONs on the Mutant Allele of the *COL6A* Genes In the absence of AON, the mutant allele is transcribed and translated into a defective monomer. This affects the assembly of the collagen VI tetramer, which fails to be secreted into the ECM. Following the gapmer AON uptake by cellular endocytosis, it can hybridize with either pre-mRNA in the nucleus or mature mRNA in both the nucleus and cytoplasm. Hybridization of the gapmer AON-mutant mRNA/pre-mRNA induces the activation of RNase H, leading to selective degradation of the mutant mRNA/pre-mRNA. This strategy will retain the transcription of the wild-type allele, followed by the translation of normal collagen VI monomer and efficient assembly of a normal tetramer. The normal tetramer is eventually secreted and functions within the ECM.

Collagen VI is one of the microfibrillar components of the extracellular matrix (ECM) expressed in a wide variety of tissues and shows a particular association with basement membranes by interacting with cells indirectly via components of the basal lamina.^5^ Collagen VI undergoes an extensive assembly process inside the cell before being secreted into the ECM. The assembly begins with the formation of the basic monomer, which is composed of one of each of the three α chain subunits encoded by the three collagen 6 genes. Within the cell, these monomers associate to form dimers, which then pair up into tetramers. These tetramers are then secreted into the ECM, where they assemble in an end-to-end fashion to give rise to the final microfibrillar network.^5^, ^6^ Mutations in any of the three collagen 6 genes disrupt the correct formation of tetramers and they consequently fail to be secreted into the ECM to form collagen VI microfibrillar structures (Figure 1).^3^ The role of collagen VI in muscle is not completely understood. A role in mitochondrial survival had been previously reported.^7^ A more recent study in organizing the satellite cell niche, which regulates muscle regeneration, was also demonstrated.^8^

Bethlem myopathy is almost exclusively caused by dominant *COL6A1*, *COL6A2*, or *COL6A3* mutations, whereas UCMD occurs as a result of both recessive and dominant mutations. In populations with a low degree of consanguinity, de novo autosomal-dominant mutations are the most common underlying mechanism of UCMD.^3^, ^9^ There are two common types of dominant mutations in COL6-CMD: one is represented by in-frame deletions or splice site mutations causing in-frame exon skipping and another is represented by missense mutations. These types of mutations act in a dominant-negative fashion due to the failure in incorporating the mutated α chain subunits into collagen VI monomers. Interestingly, complete deletion of one copy of the three *COL6A* genes is not associated with any clinical phenotypes, as indicated by the parents of children affected by the recessive variant, who may carry nonsense mutations or a large genomic deletion but remain completely asymptomatic.^10^ This suggests that suppression of one allele of the *COL6A* gene is not associated with a clinical phenotype and therefore may be used as a therapeutic strategy. Specifically, it has been hypothesized that silencing the mutated allele in one of the *COL6A* genes by allele-specific antisense oligonucleotides (AONs) may correct the abnormal collagen VI expression in CMD caused by dominant mutations. The fact that dominant-negative mutations account for 50%–75% of UCMD cases^11^, ^12^ and nearly 100% of BM cases^11^, ^13^ makes an allele-specific silencing approach an interesting therapeutic strategy for a large proportion of COL6-CMD patients.

Encouraging data have been reported recently from a study using AON to target an SNP and induce out-of-frame exon skipping in the *COL6A2* gene to deplete the mutated transcript via RNA nonsense-mediated decay. The preferential skipping of the target exon via SNP recognition recovered the production of functional collagen VI in cultured fibroblasts from a patient with UCMD.^14^ This approach was further supported by two additional studies using a small interfering RNA (siRNA) approach to selectively silence the mutant allele, which carries the heterozygous exon 16 deletion in the *COL6A3* gene^15^ and the missense c.850G > A (p.G284R) mutation in the *COL6A1* gene.^16^ These studies provide the proof of principle for allele-selective suppression by different RNA-based approaches as a therapeutic strategy in COL6-CMD.

In this study, we describe the investigation of gapmer AONs for allele-specific silencing (Figure 1). Gapmer AONs were designed to selectively bind to the mutant allele to induce target RNA degradation by activating RNase H, an endonuclease that cleaves the RNA strand of a DNA-RNA duplex.^17^, ^18^ A skin fibroblast cell line from a UCMD patient carrying a de novo 18-nt genomic deletion in exon 15 of the *COL6A3* gene was selected as an in vitro model. A series of gapmer AONs were designed with various combinations in length by targeting the premature mRNA (pre-mRNA) and mRNA, respectively. Our study shows that gapmer AONs can selectively suppress the expression of the mutant transcripts while not interfering with the wild-type transcripts. Functionally, AON treatment significantly reduces the intracellular retention of the collagen VI complex and increases the secretion of a functional protein in the ECM. This is a major step forward in establishing the allele-specific silencing by AON as a therapeutic strategy for the treatment of dominant COL6-CMD.

## Results

### Gapmer AONs Designed to Bind the Deletion Site of *COL6A3* and Target Either Pre-mRNA or mRNA Products

Gapmer AONs were designed to stimulate RNA degradation through the recruitment of RNase H. In humans, the specific enzyme recruited by the AON-RNA duplex is RNase H1.^19^ It has been reported that RNase H active AONs exert their action predominantly in the nucleus, where they interact with the pre-mRNA^20^, whereas in other reports, mRNA in the cytoplasm is the predominant target of RNase H active AONs.^18^ To understand if the gapmer AONs preferentially target the pre-mRNA or mRNA of the *COL6A3* gene, we selected a fibroblast cell line carrying a specific 18-nt genomic deletion (c.6135_6152del; p.Ile2046_Pro2051del) in exon 15 of *COL6A3* as the cellular model. This 6-aa in-frame deletion is near the amino terminus of the triple helical domain and plays a strong dominant-negative effect.^6^, ^21^

**Figure 2:**
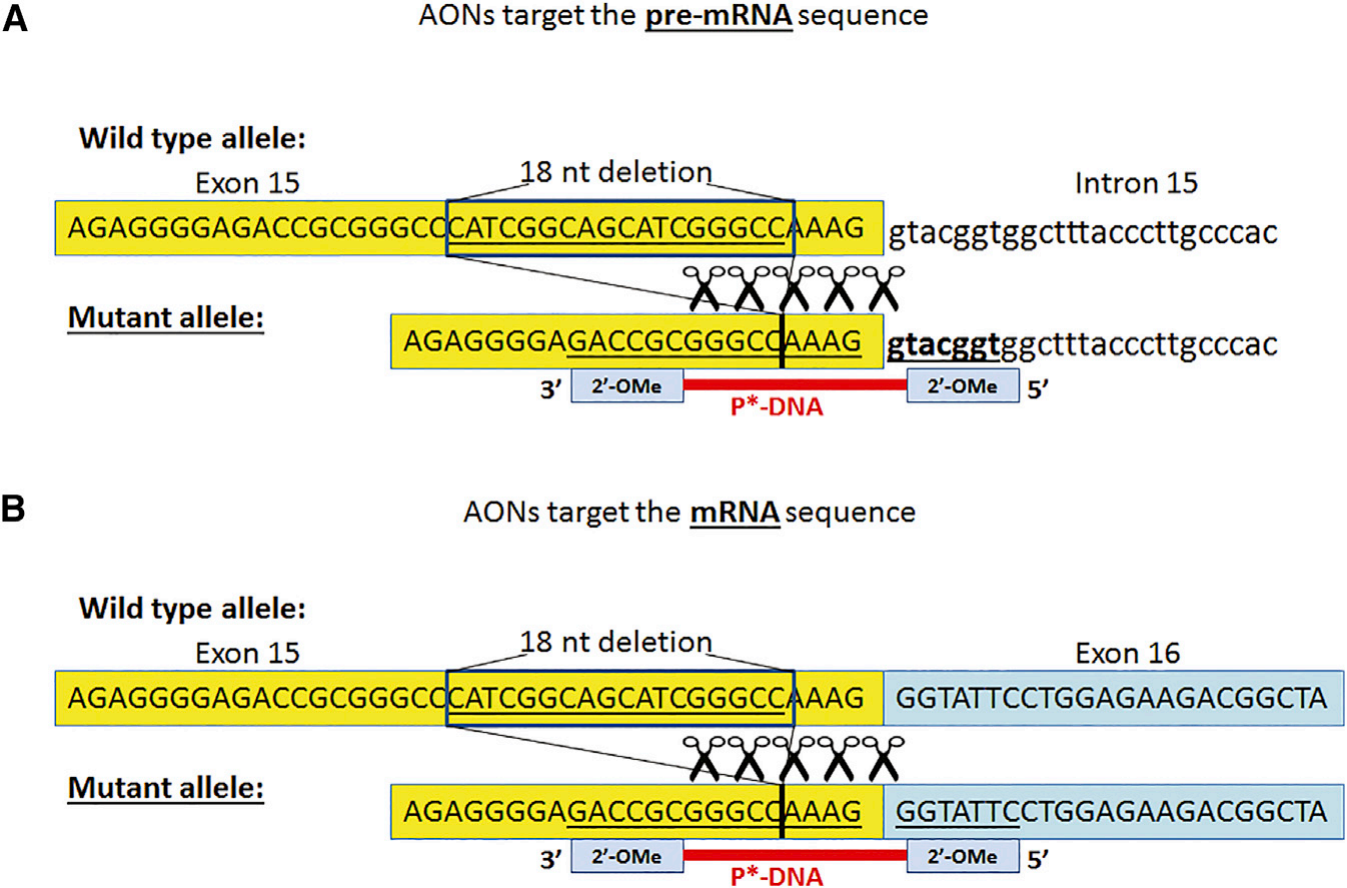
Design of Gapmer AONs Targeting the Mutant Allele An 18-nt in-frame deletion was presented in exon 15 of the *COL6A3* gene. Gapmer AONs were designed to target the mutant allele at either the pre-mRNA (A) or mRNA (B) levels. The gapmer AON was designed as a short RNase H activating phosphorothioate DNA (P*-DNA) antisense sequence flanked by short 2′-OMe RNA antisense sequence at each end. AONs targeting pre-mRNA or mRNA differ at the 5′ end with 2′-OMe chemical modification, which either binds to the conjunctive intron 15 sequence (pre-mRNA level) or exon 16 sequence (mRNA level).

Deletion of 18 nt near the 3′ end of exon 15 results in different sequences of pre-mRNA and mRNA. Therefore, unique gapmer AONs can be designed to anneal to pre-mRNA or mRNA, respectively (Figure 2). We followed the classic design of gapmer AON: the RNase H activating phosphorothioate DNA domain (DNA gap) is flanked by short RNA sequences in the 2′-OMe backbone at both ends (wings) to protect the AONs from degradation by nucleases.^22^ Two groups of gapmer AONs were designed to target either the mRNA or the pre-mRNA. Three different lengths of AONs, 16-mer, 18-mer, and 22-mer, were designed; these were associated with a different length of the DNA gap, between 6 nt and 12 nt (Table 1).

**Table 1:** AONs Tested in This Study

AON ID	AON Sequence (5′ to 3′)	Total Length (mer)	DNA Gap Length (nt)
**AON-Targeting mRNA Level**			

AON-1	[GAAUA]C*C*C*T*T*T*G*G*C*C*C*G*[CGGUC]	22	12
AON-2	[GAAUAC]C*C*T*T*T*G*G*C*C*C*[GCGGUC]	22	10
AON-3	[GAAUACC]C*T*T*T*G*G*C*C*[CGCGGUC]	22	8
AON-4	[AUACC]C*T*T*T*G*G*C*C*[CGCGG]	18	8
AON-5	[UACC]C*T*T*T*G*G*C*C*[CGCG]	16	8
AON-6	[UACCC]T*T*T*G*G*C*[CCGCG]	16	6
AON-Scr	[GAAUACA]A*T*T*T*A*A*C*C*[CUAUGUC]	22	8

**AON-Targeting Pre-mRNA Level**			

AON-7	[ACCGU]A*C*C*T*T*T*G*G*C*C*C*G*[CGGUC]	22	12
AON-8	[ACCGUA]C*C*T*T*T*G*G*C*C*C*[GCGGUC]	22	10
AON-9	[ACCGUAC]C*T*T*T*G*G*C*C*C*[GCGGUC]	22	8
AON-10	[ACCGUACC]T*T*T*G*G*C*[CCGCGGUC]	22	6
AON-11	[CGUAC]C*T*T*T*G*G*C*C*[CGCGG]	18	8
AON-12	[GUAC]C*T*T*T*G*G*C*C*[CGCG]	16	8
AON-13	[GUACC]T*T*T*G*G*C*[CCGCG]	16	6

[N], RNA in 2′-OMe backbone (wings); N*phosphorothioate (ps) DNA (DNA gap).

### Gapmer AONs Silence the Mutant mRNA Transcripts Preferentially at the mRNA Level

The difference between the wild-type transcript and the mutant transcript in this cell line is that the wild-type transcript is 18 bp longer than the mutant one when the same primers are used to amplify the PCR products flanking the mutation region. This, therefore, provides us with a practical tool to distinguish the two transcripts by gel electrophoresis. To test the efficiency of AONs on suppressing the mutant allele, we performed semiquantitative reverse transcript one-step PCR. RNA samples were isolated from the patient’s fibroblast cell line treated with different gapmer AONs at 100 nM for 24 hr. The percentage of PCR band intensity of the mutant *COL6A3* transcripts fragment (lower PCR band) to total *COL6A3* transcripts fragment (wild-type transcript/top PCR band + mutant transcript/lower PCR band) was used to measure the efficiency of mutant allele silencing.

**Figure 3:**
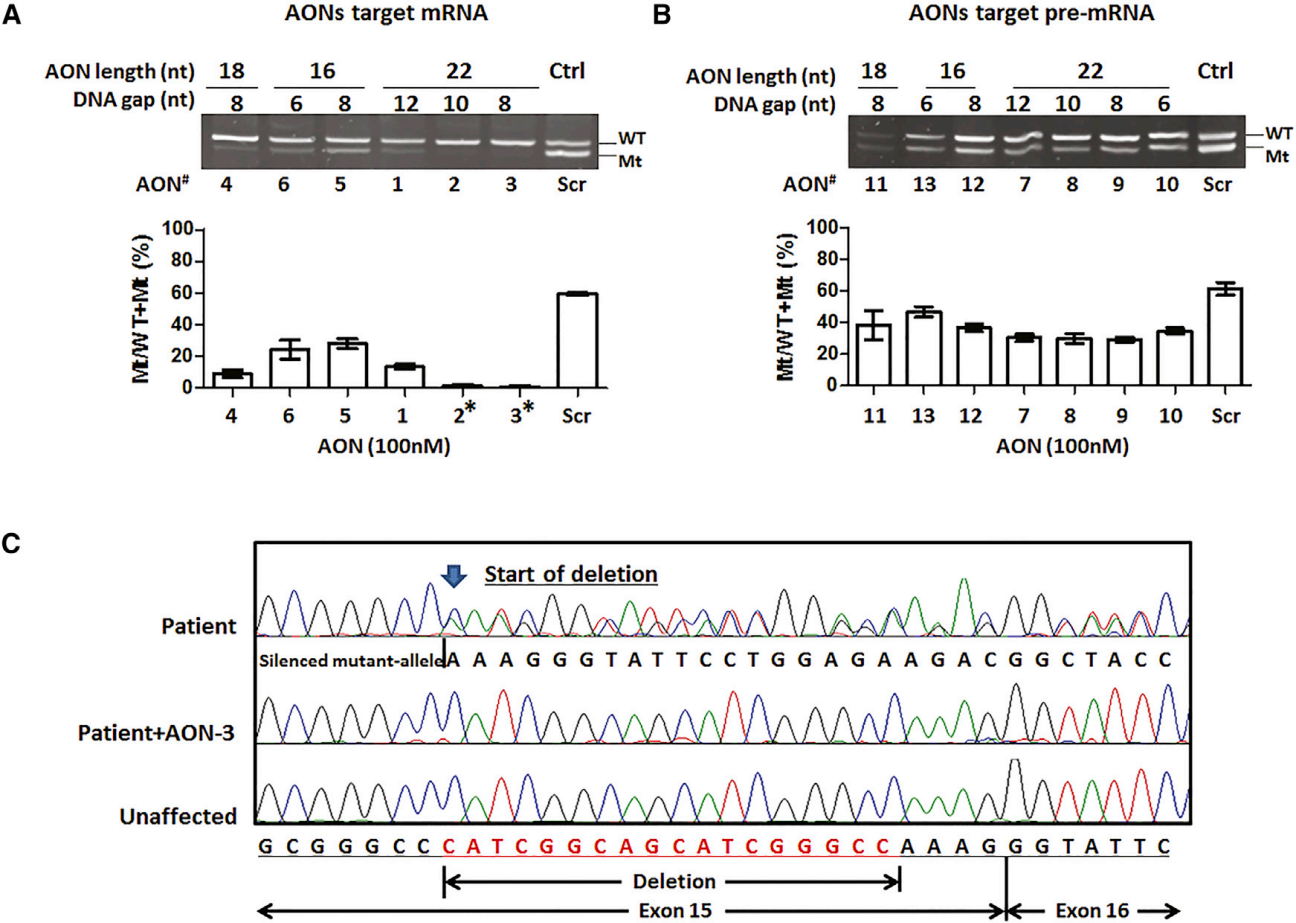
Suppression of Mutant Transcripts by Gapmer AONs A fibroblast cell line from a UCMD patient carrying the 18-nt heterozygous genomic deletion in exon 15 of *COL6A3* was treated with AONs at 100 nM for 24 hr by targeting either mRNA (A) or pre-mRNA (B) sequences. The efficiency of AONs on suppression of mutant transcripts was measured by semiquantitative one-step reverse transcript PCR. The percentage of PCR band intensity of the mutant transcripts to total transcripts (Mt/WT+Mt (%)) was used to measure the efficiency of mutant allele suppression. The AON-Scr-treated UCMD fibroblast was used as control. Measurement was performed in three individual experiments. *AON-2 and AON-3, targeting the mRNA sequences with 22-mer long and a DNA gap size of 8–10 nt, showed the most significant suppression on mutant transcripts. (C) Sanger sequencing showed the silencing of the mutant allele in patient fibroblasts after AON-3 treatment. Data are presented as mean ± SEM.

In untreated or scrambled AON-treated fibroblasts, there was approximately 60%–70% expression of mutant to total *COL6A3* transcripts. The mutant transcripts were dramatically reduced after AON treatment. Gapmer AONs suppressed the expression of mutant transcripts at both mRNA (Figure 3A) and pre-mRNA levels (Figure 3B). AONs targeting mRNA achieved more significant suppression on the mutant transcripts than those targeting pre-mRNA. In the pre-mRNA targeting group, there was approximately 25%–50% suppression of the mutant transcripts compared to the scrambled control (Figure 3B). However, in the mRNA-targeting group, the mutant transcripts were 50%–100% suppressed compared to control. The most striking suppression was detected in cells treated with the 22-mer gapmer AONs. Among them, the AONs with an 8-nt (AON-3) and 10-nt (AON-2) DNA gap had the most significant efficiency, with near complete suppression of the mutant transcripts. This was followed by the 22-mer AON with a 12-nt DNA gap (AON-1) and the 18-mer AON with an 8-nt DNA gap (AON-4), which showed approximately 80%–85% silencing of the mutant transcripts. The short 16-mer AONs with a 6- and 8-nt DNA gap (AON-6 and AON-5) also showed mutant-allele silencing, although to a lesser extent, and achieved approximately 50%–60% suppression. In addition to the semiquantitative one-step PCR, we performed Sanger sequencing of PRC products amplified in RNA samples collected from AON-3-treated fibroblasts. Complete silencing of the mutant allele and near-normal sequencing chromatogram was detected in fibroblasts after AON-3 treatment (Figure 3C).

**Figure 4:**
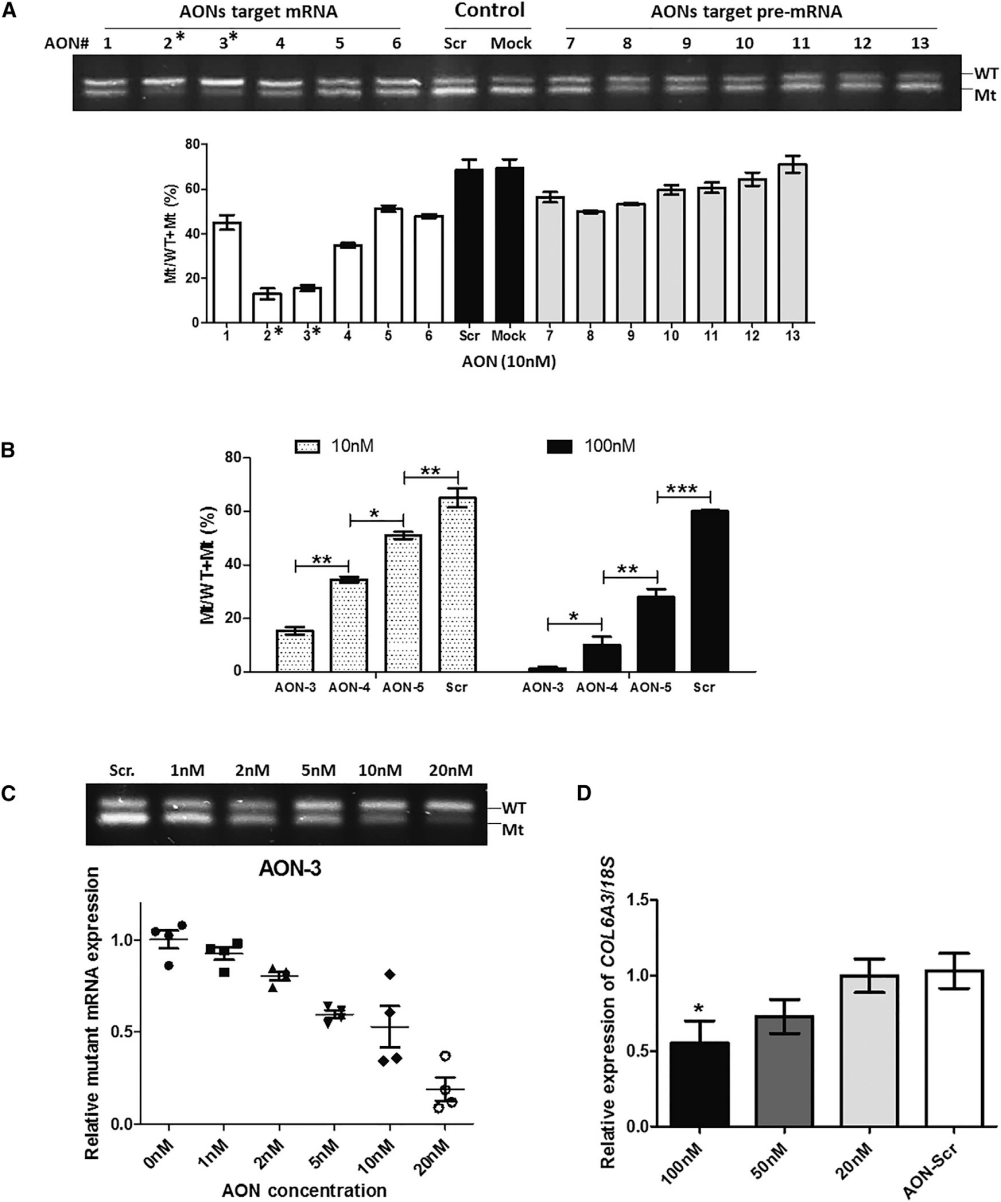
AON-3 Suppressed the Mutant Transcripts in a Dose-Dependent Manner (A) One-step reverse transcript PCR of mRNA isolated from patient fibroblasts treated with 13 AONs at 10 nM. Measurement was performed in three individual experiments (n = 3 per concentration). *AON-2 and AON-3 exhibited the most efficient suppression, over 80%, on the mutant transcripts. (B) Comparison of the efficiency of AON-3 (22-mer), AON-4 (18-mer), and AON-5 (16-mer), all with the same 8-nt DNA gap, in suppressing the mutant transcripts at concentrations of 10 nM and 100 nM, respectively. There was a significant difference between AONs with different lengths (*p < 0.05; **p < 0.01; ***p < 0.001; n = 3 per group). (C) Fibroblasts were treated with AON-3 at a range of concentrations of between 1 nM and 20 nM. The IC_50_ was identified at 5–10 nM. There was an approximately 90% reduction of the mutant transcripts in cells treated at 20 nM (n = 4 per concentration). (D) Effects of AON-3 on wild-type transcripts (n = 3 per concentration). At 100 nM, approximately 50% of wild-type *COL6A3* transcripts were affected (*p < 0.05). No transcript reduction was detected at 20 nM. Data are presented as mean ± SEM.

To further determine the efficacy of these AONs, we lowered the AON concentration to 10 nM and measured the effect on mutant-allele expression (Figure 4A). At this concentration, the superiority of mRNA-targeting AONs became more evident than pre-mRNA-targeting AONs. AON-2 and AON-3 were the most efficient AONs and showed an approximate 80% suppression on the mutant transcripts at 10 nM. By using ANOVA, we compared the efficiency of AON-3, AON-4, and AON-5, the three 22-mer, 18-mer, and 16-mer mRNA-targeting AONs with the same 8-nt DNA gap. There were significant differences in the efficiency on mutant-transcript suppression between AONs with different lengths in both the 10 nM and 100 nM AON-treated groups (Figure 4B, p < 0.0001). The 22-mer AON gave the greatest efficiency, followed by the 18-mer AON and then the 16-mer AON.

### Gapmer AONs Selectively Silence the Mutant Transcripts in a Dose-Dependent Manner

To understand the range of effective concentrations of the lead gapmer AON in silencing the mutant transcripts, we treated patient fibroblasts with AON-3 at concentrations of 1, 2, 5, 10, and 20 nM for 24 hr. A clear dose response was detected starting as low as 2 nM and reached 50% suppression (IC_50_) at concentrations between 5 nM and 10 nM. A reduction of approximately 90% of the mutant transcripts was detected in cells treated at 20 nM (Figure 4C).

To exclude the possible suppression on the wild-type transcripts by AONs, a potential undesirable off-target effect, we examined the effect of AON-3 on *COL6A3* transcripts in fibroblasts from a healthy donor. Control fibroblasts were treated with AON-3 at 20, 50, and 100 nM for 24 hr. Quantitative reverse transcript real-time PCR was performed to measure the abundance of the wild-type *COL6A3* transcripts (Figure 4D), normalized to endogenous 18S transcripts. At 100 nM, *COL6A3* wild-type transcripts were reduced by approximately 50% (p < 0.05). A slight reduction (∼25%) of the wild-type transcripts was also detected in 50 nM AON-3 treated cells, although this was not statistically significant. No transcript reduction occurred in cells treated at 20 nM. We therefore chose 20 nM as the concentration for the subsequent protein studies.

### Gapmer AONs Decreased Intracellular Collagen VI Retention and Increased Collagen VI Deposition in the ECM in UCMD Fibroblasts

**Figure 5:**
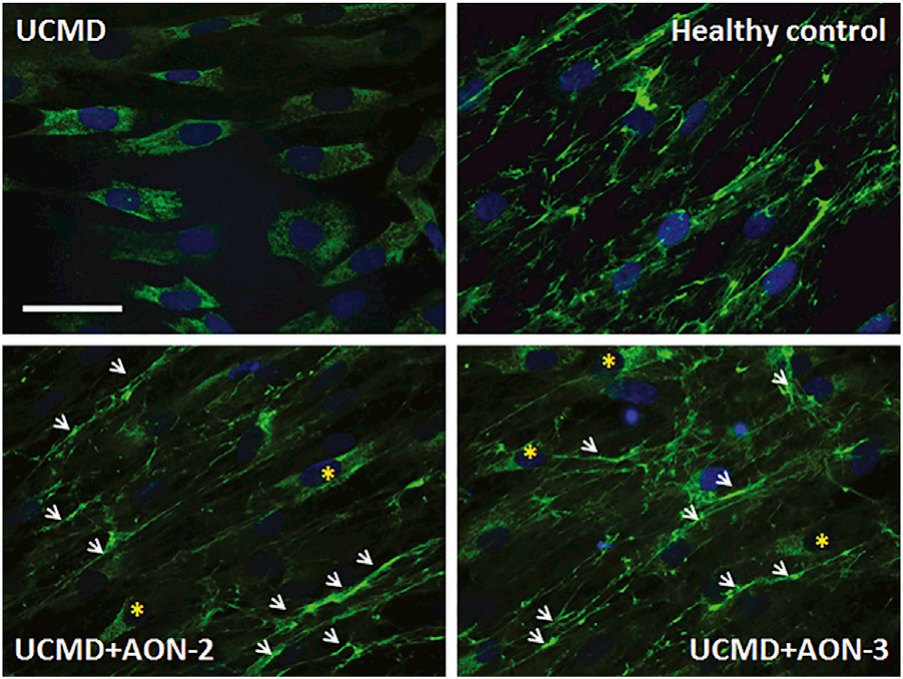
Gapmer AON Decreased Intracellular Collagen VI Retention and Increased Its ECM Deposition in Fibroblasts The fibroblasts were permeabilized by 0.1% Triton in blocking buffer during immunostaining. Strong intracellular accumulation was observed in patient’s untreated fibroblasts (UCMD) compared to healthy control fibroblasts, where collagen VI was localized in the ECM. After a single treatment of AON-2 (UCMD+AON-2) or AON-3 (UCMD+AON-3) at 20 nM, the retention of intracellular collagen VI was markedly reduced, although it was notable occasionally in some cells (asterisk) and accompanied by partial deposition in the ECM (arrow).

Significant accumulation of intracellular collagen VI is a typical feature in cultures of UCMD fibroblasts.^23^ The UCMD fibroblasts used in this study displayed strong intracellular collagen VI retention, detected when collagen VI protein was stained on permeabilized cells treated by 0.1% Triton in blocking buffer (Figure 5). Patient fibroblasts were treated with 20 nM AON-2 and AON-3 for 24 hr in transfection medium and then replaced with growth medium with 50 μg/mL ascorbic acid for another 24 hr before being processed for immunostaining. The retention of intracellular collagen VI was markedly reduced and accompanied by increased collagen VI protein deposition in the ECM (Figure 5).

**Figure 6:**
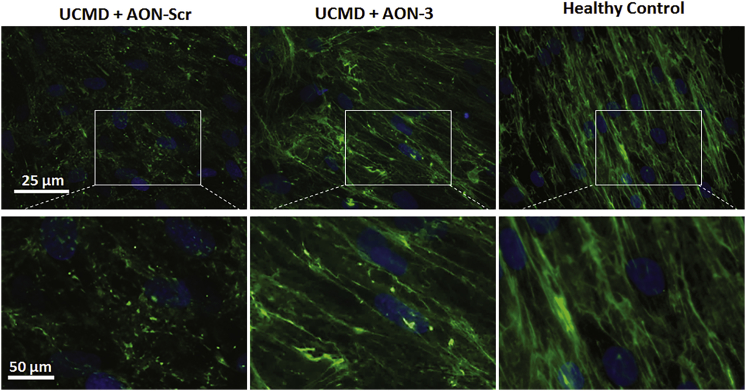
Effect of AON-3 on Collagen VI Deposition in the ECM Representative images of collagen VI α3 staining (in green) in unpermeabilized fibroblasts treated with scrambled AON (UCMD+AON-Scr), AON-3 (UCMD+AON-3), and healthy control fibroblasts. Higher magnification of the images is presented in the lower panel. Scale bar, 25 μm and 50 μm, respectively.

In unpermeabilized cells, where immunofluorescence staining only detects collagen VI in the ECM, collagen VI fibrils were dramatically reduced in patient fibroblasts and replaced by a discontinuous and speckled staining pattern (Figure 6). After a single AON-3 treatment at 20 nM, the pattern of collagen deposition changed to linear fibrils, similar to the pattern in healthy control fibroblasts (Figure 6).

**Figure 7:**
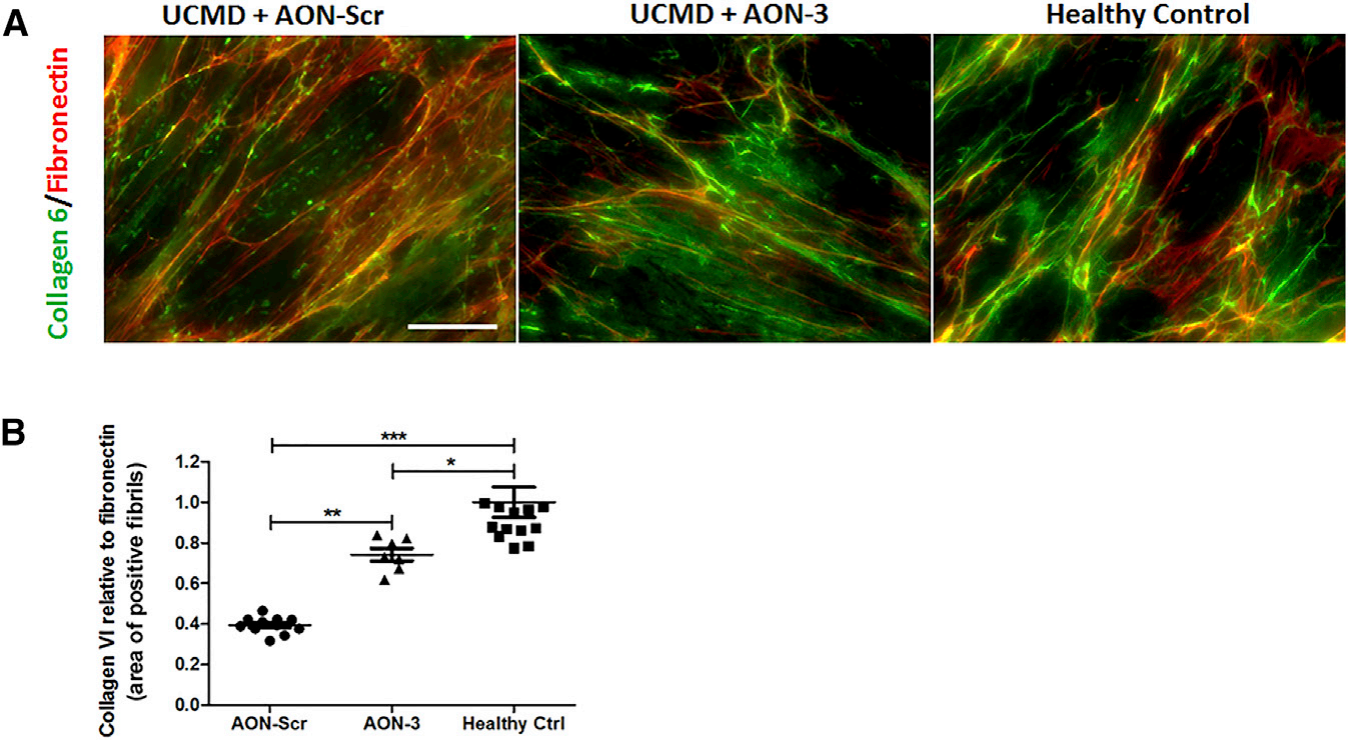
Gapmer AON Increased ECM Deposition of Collagen VI in Co-staining with Fibronectin in Fibroblast (A) Double immunostaining of collagen 6 (green) and fibronectin (red) was performed in a patient’s unpermeabilized fibroblasts treated with scrambled AON (UCMD+AON-Scr) and AON-3 (UCMD+AON-3) and compared with staining in fibroblasts from a healthy control. Scale bar, 30 μm. (B) Semiquantification of the fluorescence signals from collagen 6, relative to the signals from fibronectin, was performed using ImageJ software. Three to four images were captured from each coverslip, and the experiment was performed in triplicate (*p < 0.05; **p < 0.01; ***p < 0.001; n = 9–12 images/group). Data are presented as mean ± SEM.

Fibronectin is another main component of the ECM and associates with collagen VI in cultured fibroblasts, although the two microfibrillar systems do not completely co-localize.^24^ To examine the effect of AON treatment on the organization of collagen VI and fibronectin in UCMD fibroblasts, we performed double staining of collagen VI and fibronectin in the unpermeabilized cells (Figure 7A). Studies in *Col6a* null mice and CMD patient fibroblast cultures showed that fibronectin levels and expression were similar to controls and indicates that the synthesis and secretion of fibronectin is not dependent on collagen VI synthesis or its secretion in the culture medium.^24^ We therefore performed semiquantitative fluorescence imaging analysis of ECM collagen VI abundance and used fibronectin as a control ECM protein marker. Images were analyzed and quantified using ImageJ software. The analysis of the area of collagen-VI-positive fibrils relative to that of fibronectin-positive fibrils was compared between scrambled and AON-3-treated patient fibroblasts and healthy control fibroblasts. There was a strong reduction in ECM collagen VI abundance in the scrambled AON-treated patient’s fibroblast compared to the healthy control (p < 0.0001), and was significantly increased after AON-3 treatment (p < 0.01), although the expression was still below the level of the healthy control (p < 0.05) (Figure 7B).

**Figure 8:**
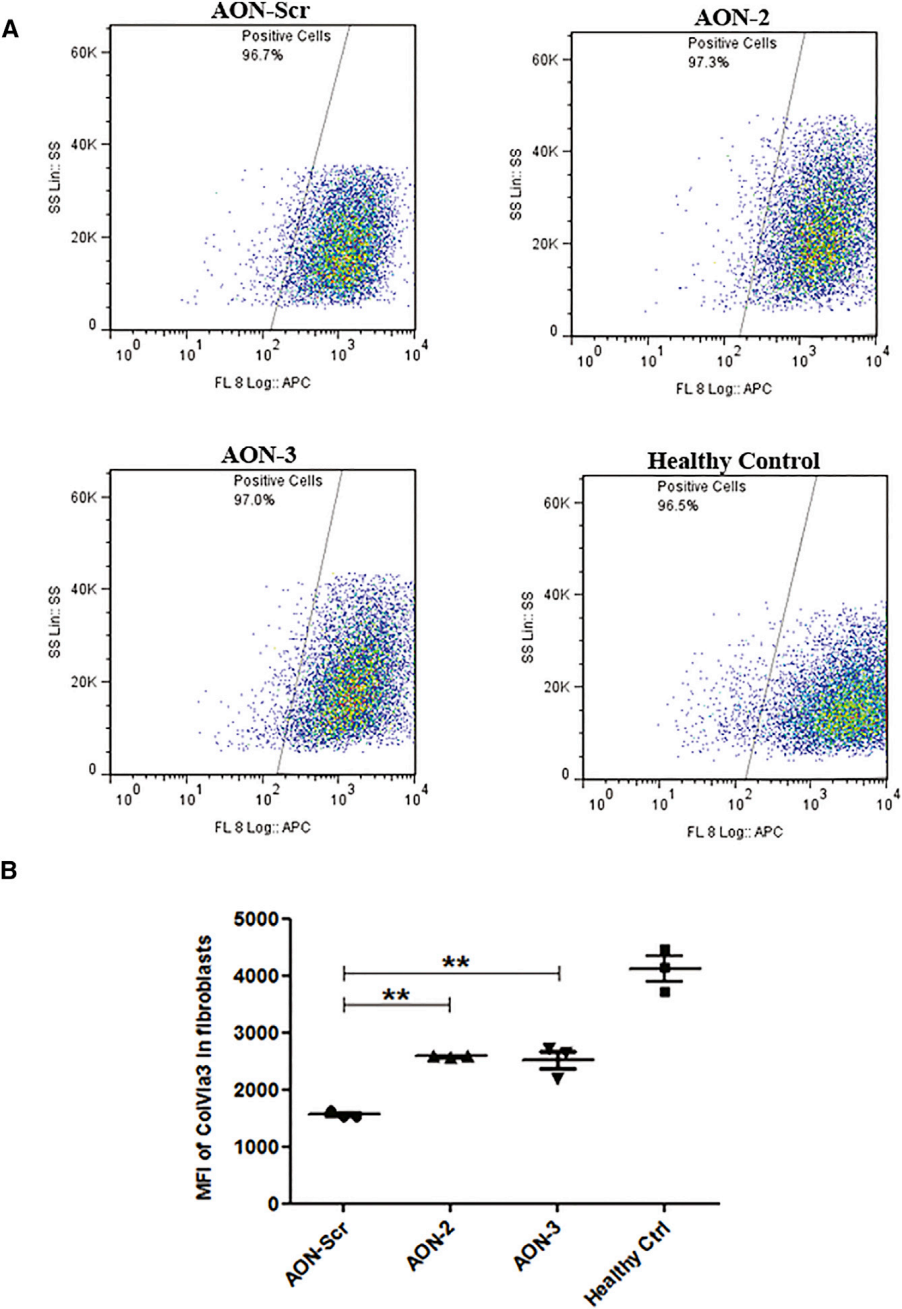
Quantification of ECM Collagen VI α3 Protein in Fibroblasts by Flow Cytometry (A) Representative flow cytometry images of collagen VI α3 analysis. Flow cytometry analysis was performed in patient fibroblasts treated with AON-Scr, AON-2, and AON-3 and healthy control fibroblasts. A total of 10,000 cells were collected and gated using the side scatter on the y axis and fluorescence intensity of collagen VI α3 immunolabeling (FL8 log-APC) on the x axis. (B) The MFI of collagen VI α3 in fibroblasts treated with scrambled control AON (1,558 ± 37.16; n = 3) was dramatically reduced compared to the MFI in healthy control cells (4,107 ± 222.9; n = 3; p < 0.0001). This was significantly increased after AON-2 (2,582 ± 13.58; n = 3) and AON-3 (2,516 ± 160.4; n = 3; **p < 0.01) treatment. Data are presented as mean ± SEM.

To quantitatively determine the change of ECM collagen VI, we performed flow cytometry of collagen VI α3 in cultured fibroblasts.^25^ The standard operating procedure of the collagen VI flow cytometry assay used in the diagnostic services in our laboratory is a 5-day procedure, starting from fibroblast seeding to final cell harvest for detection. Considering the longer procedure time (5 days) than immunohistochemistry (2 days), we therefore performed two consecutive transfections in fibroblast cultures. Patient fibroblasts received two 20 nM AON-2, AON-3, or scrambled AON (AON-Scr) treatments at 48-hr intervals and 50 μg/mL L-ascorbic acid for 24 hr on the last day before being processed for the flow cytometry assay. The mean fluorescence intensity (MFI) in the scrambled AON-treated fibroblast (1,641 ± 35.69; n = 3) was dramatically reduced compared to the healthy control (4,117 ± 222.8; n = 3; p < 0.0001). This equated to 40% of healthy control levels. The MFI in patient fibroblasts was significantly improved after AON-2 (2,653 ± 11.17; n = 3; p < 0.001) and AON-3 (2,570 ± 155.7; n = 3; p < 0.001) treatment to approximately 60% of the healthy control levels (Figure 8).

## Discussion

In this study, we describe for the first time the development of an allele-specific silencing approach using gapmer AONs to diminish the dominant-negative effect in a UCMD fibroblast cell line carrying a dominant mutation in the *COL6A3* gene.

Gapmer is a chimeric AON that contains a central sequence of phosphorothioate DNA nucleotides (“DNA gap”) flanked by sequences of modified RNA residues at each end to protect the DNA gap from nuclease degradation, whereas the central DNA gap region allows RNase-H-mediated cleavage of the target RNA.^26^ RNase-H-mediated RNA degradation by gapmer AONs is a commonly used strategy to regulate gene expression. Increasing numbers of second generation gapmer AONs, with 2′-*O*-modifications (i.e., 2′-*O*-methoxy-ethyl and 2′-OMe), have proceeded to different phases of clinical trials in the last decade and have expanded to many disease fields (see review^27^, ^28^, ^29^, ^30^). RNase H is a ubiquitous enzyme found in both the nucleus and cytoplasm of all cells. Although the majority of the intracellular RNase H is localized in the nucleus,^31^ far lower but still detectable amounts (∼5%) are present in the cytoplasm.^32^ The relative contribution of RNase H in these two different cellular compartments is still unclear. Observations that gapmer AONs efficiently silence gene expression by targeting intron sequences,^20^ which are assumed to be only found in the nucleus, or nuclear RNA^33^, ^34^ and nuclear long non-coding RNA (lncRNA),^35^ suggest that the nucleus is the major cellular compartment where gapmer AONs have effect. Activity in the cytoplasm is less clear. However, a recent report of a cytoplasmic pathway for gapmer AON-mediated gene silencing in mammalian cells provides a new mechanism^36^ and suggests that nuclear targeting is not an absolute requirement for gene silencing.

In this study, we designed two groups of gapmer AONs that targeted mRNA and pre-mRNA sequences, respectively. Our study showed that although both groups presented efficiency in allele-specific silencing, the AONs targeting mRNA levels are superior to those targeting pre-mRNA levels (Figure 3). In this case, it is possible that both nucleus and cytoplasmic RNase H mechanisms are involved in this process. The differential efficiencies between AONs targeting exon and intron sequences have also been reported previously in other genes.^20^ Possible reasons why AONs targeting mRNA sequences work better than pre-mRNA sequences in the *COL6A3* gene may include (1) the difference in the secondary structures of the two sequences; (2) different uptake efficiency of AONs in different cellular compartments; and (3) cellular localization of target transcripts. The secondary structure of the target mRNA or pre-mRNA is an important determinant of activity for all RNA-related therapies, including siRNA and AONs for gene silencing and splicing modulation. It is assumed that the predicted secondary structure of the mutant allele at the pre-mRNA level containing intron sequences is different from the structure of mature mRNA in this case. The cellular uptake of AON is predominantly mediated by endocytosis. AONs then continuously shuttle between cytoplasm and the nucleus through yet an unidentified cellular pathway with molecules that undergo nucleo-cytoplasmic transport.^37^ Understanding of the pathways that result in efficient AON trafficking to different intracellular active compartments, and especially between cytoplasm and nucleus, will help to figure out the difference observed above between the two AON target groups. It is thought that mature protein-coding mRNAs predominantly reside in the cytoplasm for subsequent protein synthesis, whereas pre-mRNA exclusively resides in the nucleus. It is therefore possible that the abundant *COL6A3* transcripts in the cytoplasm of the cell line in our study may influence the gapmer AON activities.

To elicit mRNA degradation by RNase H, a gap of at least 5 consecutive phosphorothioate DNA nt, with 7–10 being optimal, is usually incorporated into the design of gapmer AON.^26^, ^38^ In this study, we compared a number of different lengths of the gapmer AON sequences (16-, 18-, and 22-mer), and the central DNA gap (6, 8, 10, and 12 nt). Our findings showed that the best antisense activity was found with 22-mer gapmers and a DNA gap size of between 8 and 10 nt (Figures 3 and 4). Length of AONs is also an important parameter in determining the antisense activities in different applications.^39^, ^40^ Gapmer AONs are typically composed of 16–22 nt for general gene silencing. Usually, the longer the sequence, the stronger the binding affinity. However, in the case of allele-specific gene silencing, a long AON sequence may reduce its specificity in discriminating the mutant and wild-type alleles. Therefore, calibrating the length of AONs and the size of the DNA gap is required in the design of allele-specific silencing gapmer AONs.

The effective concentration (IC_50_) of the lead gapmer AON determined in this study was as low as 5–10 nM (Figure 4C). An undesirable and significant off-target effect on the wild-type transcripts was detected at the higher concentration of 100 nM. Although it is 10-fold higher than the IC_50_, the possibility of adverse events related to the targeting of the wild-type sequence by the gapmer AON needs to be taken into consideration when evaluating future in vivo studies. Newer generations of oligonucleotide chemical modifications may be applied to overcome this limitation of gapmer AON in the 2′-OMe backbone.

Further chemical modifications of the sugar at the 2′ position, such as 2′-OMe, 2′-O-methoxy-ethyl (2′-MOE), and locked nucleic acids (LNAs), improve the RNA-binding affinity, increase resistance to the endonuclease, decrease toxicity, and prolong tissue half-life and preserve RNase H activation to the RNA:AON duplex. LNA gapmer AONs with two or three LNA moieties placed at the 3′ and 5′ ends have been widely used in gene silencing.^38^ However, the striking acute hepatotoxicity induced by some LNA gapmer AONs in mice in preclinical studies have limited the clinical development of this chemical modification.^41^ In contrast, 2′-MOE gapmer AONs have a more favorable safety profile, as suggested by a number of clinical studies.^42^ Moreover, systemically administered MOE gapmer AONs, when targeting transcripts with prolonged nuclear residence, can enter skeletal muscle fibers and cause rapid target gene knockdown via RNase H1 in the transgenic mouse model of myotonic dystrophy.^34^ Gapmer AONs with another sugar modification, such as the 2′-deoxy-2′-fluoro-β-D-arabinonucleic acid (2′F-ANA), have also improved RNase H activity and reduced cell toxicity.^18^, ^43^ We only measured the cellular activity of gapmer AON with the 2′-OMe modification in this study. Investigations of more chemical modifications, such as MOE and 2′F-ANA, may provide more efficient candidates for the allele-specific silencing approach in COL6-CMD.

Because interstitial fibroblasts are the main source of collagen 6 synthesis in skeletal muscle,^44^ the uptake and efficiency of gapmer AONs in skeletal-muscle-originated interstitial fibroblasts is yet to be elucidated. Although modified AONs such as MOE, LNA, 2′F-ANA, and other emerging ones may improve the biodistribution, advanced technology, such as cell-penetrating peptide-mediated oligonucleotide therapy, may also provide further benefit.^45^

Allele-specific knockdown using siRNA has been reported to efficiently and selectively silence the mutant mRNA transcripts in UCMD with dominant mutations in *COL6* genes, such as the heterozygous exon 16 deletion in the *COL6A3* gene,^15^ and the missense c.850G > A (p.G284R) mutation in the *COL6A1* gene.^16^ Treatment with lead siRNAs reduced intracellular collagen VI retention and increased the quantity and quality of collagen VI microfibrils assembled in the ECM.^15^ Although siRNA is potent and effective in gene silencing in in vitro cell-based assays, hurdles such as the poor in vivo stability of the unmodified nucleotides, passive delivery and ineffective uptake to target cells, potential off-target effects, and immune stimulation have hampered its translation from the bench to further clinical application.^18^ Approaches, including chemical modifications of the RNA bases and packaging into advanced in vivo delivery cargos and an alternative form of short hairpin RNAs (shRNAs) with viral vectors, may be applied to improve the stability and uptake of siRNA in vivo.

AON technology offers great potential for conditions that cannot be impacted by traditional drugs. This technology has been applied as experimental therapies in a number of neuromuscular disorders by targeting different modulatory mechanisms in RNA processing and has shown tremendous potential in treating these otherwise untreatable diseases.^46^ The two very recent accelerated approvals by the Food and Drug Administration (FDA) of Eteplirsen, a phosphorodiamidate morpholino AON that modulates splicing in the *Dystrophin* gene to treat patients with Duchenne muscular dystrophy,^47^, ^48^ and Nusinersen, an MOE AON that modulates splicing in the *SMN2* gene to treat patients with spinal muscular atrophy,^49^, ^50^ are a huge step forward in facilitating AONs as therapeutic strategies in genetic diseases and will undoubtedly promote similar development in other neuromuscular diseases. Our group and others have provided strong and promising proof-of-concept studies on allele-specific silencing as a therapeutic strategy for COL6-CMD by investigating various RNA-related approaches. Future studies on the efficacy of gapmer AON-induced allele-specific silencing in vivo in animal models are required prior to its translation to clinical evaluation.

In conclusion, we have designed and critically evaluated gapmer AONs to elicit allele-specific silencing on dominant mutation in the *COL6A3* gene in patient fibroblasts. Gapmer AONs targeting mRNA sequences, with a length of 22-mer and a DNA gap size of 8–10 nt gave the most efficient allele-specific silencing effect. The effective concentration can be as low as 5–10 nM, whereas a high concentration is associated with off-target effects. This design principle can be applied to target a broad range of mutations with dominant-negative effects in any *COL6A1*, *COL6A2*, and *COL6A3* genes. This AON approach may benefit more than 50% of UCMD and nearly all Bethlem myopathy patients that are affected by dominant mutations in one of three *COL6A* genes. The next step will be to validate gapmer AONs in different chemical modifications and in vivo in the relevant mouse model and eventually translate to clinical studies.

## Materials and Methods

### *COL6A3* Mutation and AON Design

The mutation in the UCMD patient whose fibroblasts were used for this study is a heterozygous 18-nt genomic deletion in exon 15 of the *COL6A3* gene (c.6135_6152del; p.Ile2046_Pro2051del). This de novo in-frame 6-aa deletion has been confirmed at both genomic DNA and mRNA levels. A series of gapmer AONs were designed to target the mutation and its flanking sequences at both pre-mRNA and mRNA levels (Table 1). The RNase H activating ps DNA domain (DNA gap) is flanked by short RNA sequences in the 2′-OMe backbone at the 5′ and 3′ end to protect the AONs from degradation by nucleases. Lengths ranging from 16 to 22 bases for the entire sequence and 6 to 12 bases for the DNA gap were designed and analyzed in the study.

### Cell Culture and Transfection

A fibroblast cell line was established from a skin biopsy of the patient with written informed consent of parents. The Declaration of Helsinki protocols were followed. The study was approved by the Berkshire Research Ethics Committee (REC reference 05/MRE 12/32). Fibroblasts were supplied by the MRC Center for Neuromuscular Diseases Biobank London (REC reference number 06/Q0406/33, http://www.cnmd.ac.uk). Fibroblasts were cultured in growth medium, DMEM supplemented with 10% fetal bovine serum (FBS) and 1% glutamax, at 37°C and 5% CO_2_. Cells were seeded into six-well plates at a concentration of 2 × 10^5^ cells per well, which gives 90% confluence when transfected on the next day. For the immunofluorescence study, a 12-mm diameter glass coverslip pre-coated with collagen (BD Discovery) was placed in the culture dish before cell seeding. Lipofectamine 2000 (Life Technologies) was used as the transfection reagent to complex AONs in Opti-MEM (Life Technologies) according to the manufacturer’s instructions.

### Reverse-Transcription One-Step PCR and Gel Analysis

For studies at the RNA level, fibroblasts were treated with gapmer AONs at final concentrations ranging from 1 to 100 nM in transfection medium. Cells were harvested 24 hr post-transfection for RNA extraction. Total RNA from the cultured cells was extracted with the RNeasy kit (QIAGEN). The concentration of total RNA was measured by the NanoDrop spectrophotometer (Thermo Scientific). The amount of 100 ng of total RNA was used in one-step reverse-transcription PCR (QIAGEN) to amplify the transcripts. The primers (forward: 5′-TGG GCA GAG GGG AGA C-3′ and reverse: 5′-TCT TCT CCA GGA ATA CCC TTT-3′) were used to amplify the wild-type allele (82 bp) and mutant allele (64 bp). A 240-bp PCR product was also amplified for Sanger sequencing, with forward primer: 5′-CCC TGA GGC TTA ACT TGC TG-3′ and reverse primer: 5′-AAA CCT TGA GTG CCG TTC AC-3′. The products were amplified semiquantitatively as follows: a reverse-transcription reaction at 50°C for 30 min and the initial PCR activation step at 95°C for 15 min. These were followed by three-step cycling as denaturation at 94°C for 30 s, annealing at 60°C for 30 s, and extension at 72°C for 30 s, for a total of 26 cycles, and finished at 72°C for 10 min as the final extension. PCR samples were run on a 3% (for the 64- and 82-bp products) or 2% (for the 240-bp product for sequencing) agarose gel and visualized under the Gel Doc XR imaging system (Bio-Rad). The intensity of each PCR band was quantified using ImageJ software (https://imagej.nih.gov). Sanger sequencing service was provided by Source BioScience.

### Quantitative Reverse-Transcript Real-Time PCR

500 ng of RNA was used for first-strand cDNA synthesis with a Superscript III reverse transcription kit (Life Technologies). Quantitative real-time PCR was performed using the Takyon Rox SYBR qPCR kit (Eurogentec). The primer set, forward 5′-CAT CGG CAG CAT CGG-3′ (in exon 15) and reverse 5′-AAA CCT TGA GTG CCG TTC AC-3′ (in exon 17), was used for the amplification of a 117-bp product of the wild-type *COL6A3* gene in healthy control fibroblasts. PCR and analysis were performed with StepOne real-time PCR systems (Applied Biosystems) using the recommended program: activation at 95°C for 3 min, 40 cycles of denaturation at 95°C for 3 s, and annealing extension at 60°C for 30 s. Human 18S was used as the reference gene. Quantification was based on concurrent standard curves produced from serial dilutions of cDNA from the untreated healthy control fibroblasts. The cycle at which the amount of fluorescence was above the threshold (Ct) was detected. The ratios of wild-type *COL6A3* to *18S* of the treated samples were normalized, taking the ratio of the untreated sample as 1.0.

### Immunofluorescence Staining

24 hr after AON transfection, the culture medium was changed to growth medium containing 50 μg/mL ascorbic acid for another 24 hr. Cells on coverslips were then fixed with 4% paraformaldehyde for 10 min at room temperature and rinsed in PBS. After 1-hr blocking in 5% goat serum in PBS, coverslips were incubated for 1 hr at room temperature with the following primary antibodies: rabbit anti-collagen VI alpha3 chain-specific antibody (HPA010080; dilution 1:50, Sigma-Aldrich) and mouse anti-fibronectin antibody (F0791; dilution 1:100, Sigma-Aldrich). For intracellular collagen VI staining, 0.1% Triton was added in the blocking buffer for cell permeabilization. Coverslips were washed three times in PBS and then incubated with secondary antibodies (goat anti-mouse Alexa-594 and goat anti-rabbit Alexa 488; 1:500 dilution; Life Technologies) in PBS for 1 hr at room temperature. Coverslips were washed three times and nuclei were stained with Hoechst 33258 (Promega) for 5 min. Coverslips were mounted with Fluoromount (Southern-Biotech) and viewed under the Leica DMR fluorescence microscope. Images were captured digitally using Metamorph software (Universal Imaging), with fixed setting on exposure for all the slides. The semiquantification of ECM collagen VI expression in immunofluorescence staining was analyzed by ImageJ software, normalized to fibronectin ECM expression captured in the same image.

### Flow Cytometry Analysis

1.0 × 10^6^ fibroblasts were seeded in 75 cm^2^ tissue culture flasks (BD) in growth medium to achieve 90% confluence on the next day. Cells were transfected with AONs at 20 nM. 24 hr after this, the medium was replaced with growth medium for another 24 hr. This was then followed by the second 24-hr AON transfection, and medium was changed to growth medium for a further 24 hr. 50 μg/mL ascorbic acid was added for the final 24-hr incubation. Cells were detached using a non-enzymatic cell dissociation solution (Sigma), washed twice with PBS (Mg^2+^ and Ca^2+^ free, Life Technologies), and centrifuged for 5 min at 500 × *g*. Cells were fixed by incubating with 2% paraformaldehyde in PBS for 15 min on ice. Following fixation, cells were washed three times with PBS supplemented with 0.1% FBS and centrifuged at 1,850 × *g* for 4 min. Cell pellets were re-suspended and incubated with the polyclonal rabbit anti-collagen VI α3 chain-specific antibody (HPA010080; dilution 1:20, Sigma) diluted in PBS/0.1% FBS for 30 min on ice, washed twice in PBS/0.1% FBS, and centrifuged at 1,850 × *g* for 4 min. For a negative control in each group, cells were incubated with an equivalent amount of 0.1% FBS/PBS without the primary antibody. Cells were then incubated with donkey anti-rabbit immunoglobulin G (IgG) conjugated to Alexa Fluor 647, (A-31573; dilution 1:100; Life Technologies) diluted in PBS/0.1% FBS for 20 min on ice, washed twice in PBS/0.1% FBS, and centrifuged at 1,850 × *g* for 4 min. Cell pellets were re-suspended in PBS and transferred to FACS tubes (BD Biosciences). Cells were immediately analyzed using the CyAn ADP Flow Cytometer equipped with a 488-nm blue laser and a 635-nm red diode (Beckman Coulter). A total of 10,000 cells were acquired and subsequently analyzed using FlowJo software version 7.6.5 (Tree Star). The negative control was used to set up the collagen VI gate and remove the level of background fluorescence. The amount of collagen VI was assessed by the MFI of ECM collagen VI α3 expression.

### Statistical Analysis

GraphPad Prism 5.0 software was used for statistical analysis and graph design. Data were presented as mean ± SEM. One-way ANOVA and post Bonferroni test were used to determine statistical significance when comparing more than two groups. Student’s t test was used for statistical analysis of two groups of data.

## Author Contributions

E.M., P.A., and H.Z. conducted the experiments; H.Z. and F.M. designed the experiments and wrote the paper.

## Conflicts of Interest

The authors declare no conflicts of interest.
